# Mepolizumab improves clinical outcomes in patients with severe asthma and comorbid conditions

**DOI:** 10.1186/s12931-021-01746-4

**Published:** 2021-06-07

**Authors:** Peter G. Gibson, Charlene M. Prazma, Geoffrey L. Chupp, Eric S. Bradford, Mark Forshag, Stephen A. Mallett, Steve W. Yancey, Steven G. Smith, Elisabeth H. Bel

**Affiliations:** 1 School of Medicine and Public Health, University of Newcastle, Newcastle, Australia; 2 Respiratory Medical Franchise, GSK, Research Triangle Park, NC USA; 3 Yale Center for Asthma and Airways Disease (YCAAD), Yale School of Medicine, New Haven, CT USA; 4 Respiratory Therapeutic Area, GSK, Research Triangle Park, NC USA; 5 Clinical Statistics, GSK, Stockley Park, Uxbridge, Middlesex, UK; 6 Amsterdam University Medical Center, Location AMC, University of Amsterdam, Amsterdam, The Netherlands; 7 GSK, 5 Moore Drive, PO Box 13398, Research Triangle Park, NC 27709-3398 USA

**Keywords:** Mepolizumab, Severe eosinophilic asthma, Comorbidities, Upper respiratory, Cardiovascular, Treatable traits

## Abstract

**Background:**

Comorbidities can complicate the management of severe asthma; therefore, the presence of comorbid conditions or traits often need to be considered when considering treatment options for patients with severe asthma. The aim of this analysis is to investigate the efficacy of mepolizumab in patients with severe eosinophilic asthma and comorbidities.

**Methods:**

This was a post hoc analysis (GSK ID:209140) of data from the Phase IIb/III studies DREAM, MENSA, SIRIUS, and MUSCA. Patients aged ≥ 12 years with severe eosinophilic asthma were randomized to: mepolizumab 750, 250, or 75 mg intravenously or placebo (DREAM); mepolizumab 75 mg intravenously or 100 mg subcutaneously or placebo (MENSA); or mepolizumab 100 mg subcutaneously or placebo (SIRIUS and MUSCA) every 4 weeks for 24 weeks in SIRIUS and MUSCA, 32 weeks in MENSA or 52 weeks in DREAM. In this analysis the primary endpoint was the annual rate of clinically significant exacerbations; secondary endpoints were Asthma Control Questionnaire-5 score, St George’s Respiratory Questionnaire total score, and pre-bronchodilator forced expiratory volume in 1 s at study end. Subgroups were based on comorbidities at baseline.

**Results:**

Overall, 1878 patients received placebo (n = 689) or mepolizumab (n = 1189). Across all comorbidity subgroups mepolizumab reduced the rate of clinically significant exacerbations by 44–68% versus placebo, improved Asthma Control Questionnaire-5 score by 0.27–0.59 points, and improved St George’s Respiratory Questionnaire total score by 5.0–11.6 points. Pre-bronchodilator forced expiratory volume in 1 s was improved by 27.1–286.9 mL in all but one comorbidity subgroup, the diabetes mellitus subgroup.

**Conclusions:**

Mepolizumab reduces exacerbations, and improves asthma control, health-related quality of life, and lung function in patients with severe eosinophilic asthma despite comorbid conditions, including upper respiratory conditions, psychopathologies, cardiovascular conditions, gastroesophageal reflux disease, diabetes mellitus, and obesity.

*Trial registration*: https://clinicaltrials.gov/ DREAM, MEA112997/NCT01000506; MENSA, MEA115588/NCT01691521; SIRIUS, MEA115575/NCT01842607; MUSCA, 200862/NCT02281318.

**Supplementary Information:**

The online version contains supplementary material available at 10.1186/s12931-021-01746-4.

## Introduction

Severe asthma, thought to affect 5–10% of the asthma population, is characterized by poor symptom control, frequent exacerbations, and airflow limitation, despite the regular use of maintenance therapies including multiple controllers [1, 2]. Patients with severe asthma frequently exhibit comorbid conditions or traits, which add to the burden of respiratory symptoms [3–7]. These may include primary airway conditions such as allergic rhinitis, which occurs in 55–68% of patients with severe asthma, chronic rhinosinusitis with or without nasal polyposis, occurring in 45–50% of patients with severe asthma, and vocal cord dysfunction, which affects 19–50% of the severe asthma population [8]. Other comorbid conditions are also common and include gastroesophageal reflux disease (GERD), which affects 46–63% of patients with severe asthma, obesity, which occurs in 21–48% of patients with severe asthma, obstructive sleep apnea, seen in up to 88–96% of patients with severe asthma, and anxiety or depression, affecting 81% and 31% of the severe asthma population, respectively [8].

Comorbidities can complicate the management of severe asthma. Some, such as vocal cord dysfunction, coexist with or mimic asthma [9], while others, such as upper airway conditions, contribute to poor disease control by aggravating symptoms [10, 11]. As such, the presence of comorbid conditions may lead to under- or overtreatment with anti-asthmatic medications [12]. Additionally, comorbidities could result as adverse effects of asthma treatment, such as iatrogenic comorbidities, including obesity, osteoporosis, depression, and GERD, that are typically related to the use of systemic corticosteroids [6]. Furthermore, the presence of comorbid conditions, whether frequently associated with severe asthma, or simply common with aging, has the potential to alter the response to asthma therapy, either due to a change in asthma phenotype or an increased or less responsive airway inflammation or resultant anatomical changes (e.g. obesity) impacting mechanical functioning of the pleural cavity [13]. As the focus of severe asthma management moves increasingly towards personalized care, the role and importance of comorbid conditions is more often being recognized [8].

Severe eosinophilic airway inflammation in asthma is a clinically valid endotype associated with increased exacerbation risk [14]. It has been described as a treatable trait, since it is identifiable, measurable and treatable, allowing for targeted therapy to improve outcomes for individual patients [15]. Elevated blood eosinophil levels and a high number of severe exacerbations in the previous year are predictors of good response to anti-interleukin-5 and anti-interleukin-5 receptor α monoclonal antibodies [16].

Mepolizumab is a humanized monoclonal antibody that selectively targets interleukin-5 and is approved as an add-on treatment for patients with severe eosinophilic asthma [17, 18]. During the mepolizumab clinical development program, patients with severe eosinophilic asthma treated with mepolizumab showed consistent reductions in both clinically significant exacerbations and the need for systemic corticosteroids; improvements were also observed in lung function parameters, asthma symptom control, and health-related quality of life, compared with placebo [19–22]. Additionally, in patients with recurrent chronic rhinosinusitis with nasal polyps, mepolizumab treatment reduces the need for surgery and reduces symptom severity compared with placebo [23], and in patients with severe eosinophilic asthma and nasal polyps, mepolizumab has been shown to reduce the rate of clinically significant exacerbations compared with placebo [19–22]. Given that some comorbidities can aggravate symptoms and increase the risk of asthma exacerbations [10] or render asthma control more difficult to achieve, detailed data on the effect of mepolizumab in patients with other comorbidities are needed to determine whether the effect of mepolizumab is sensitive to presence or absence of these conditions. The aim of this post hoc meta-analysis of data from four Phase IIb/III clinical trials was to investigate the impact of mepolizumab versus placebo on clinically significant exacerbations, asthma control, and health-related quality of life in patients with severe eosinophilic asthma and comorbidities, including airway-related, airway-unrelated, and iatrogenic conditions, as determined by medical history.

## Methods

### Study design and treatment

This was a post hoc meta-analysis (GSK ID: 209140) of data from the Phase IIb/III, placebo-controlled, randomized, double-blind, parallel-group, multicenter studies, DREAM (NCT01000506), MENSA (NCT01691521), SIRIUS (NCT01691508), and MUSCA (NCT02281318), which assessed mepolizumab treatment in patients with severe eosinophilic asthma. Full details of these studies have been published previously [19–22]. In brief, patients enrolled in DREAM were randomized (1:1:1:1) to receive mepolizumab 750, 250, or 75 mg intravenously or placebo, plus standard of care (high-dose inhaled corticosteroids and another controller), every 4 weeks for 52 weeks. Patients enrolled in MENSA were randomized (1:1:1) to receive mepolizumab 75 mg intravenously, mepolizumab 100 mg subcutaneously or placebo, plus standard of care, every 4 weeks for 32 weeks. Patients enrolled in SIRIUS or MUSCA were randomized (1:1) to receive mepolizumab 100 mg subcutaneously or placebo, plus standard of care, every 4 weeks for 24 weeks. All four studies were conducted in accordance with the ethical principles of the Declaration of Helsinki, International Conference on Harmonisation Good Clinical Practice Guidelines, and applicable country-specific regulatory requirements [19–22].

### Patients

The four trials enrolled patients who were ≥ 12 years of age with severe eosinophilic asthma, defined as blood eosinophil count ≥ 150 cells/µL at baseline or ≥ 300 cells/µL in the prior year (or alternatively in DREAM as one of the following: a sputum eosinophil count of ≥ 3%, an exhaled nitric oxide concentration of ≥ 50 ppb, or prompt deterioration of asthma control after ≤ 25% reduction in regular maintenance inhaled or oral corticosteroids [OCS]). Additional criteria included a history of ≥ 2 exacerbations requiring systemic corticosteroids in the year prior to enrolment despite regular treatment with high-dose inhaled corticosteroids in the 12 months prior to screening, plus additional controller medication(s) for ≥ 3 months, and evidence of airflow obstruction. The SIRIUS study did not require a history of ≥ 2 exacerbations but did require a 6-month history of maintenance treatment with systemic corticosteroids (Additional file 1: Table 1).

### Endpoints and assessments

The primary endpoint of this meta-analysis was the annual rate of clinically significant exacerbations, defined as a worsening of asthma that required the use of systemic corticosteroids and/or hospitalization/emergency room visits. Exacerbations separated by less than 7 days were treated as a continuation of the same exacerbation. Secondary endpoints included changes from baseline in pre-bronchodilator forced expiratory volume in 1 s (FEV_1_), St George’s Respiratory Questionnaire (SGRQ) total score, and Asthma Control Questionnaire (ACQ)-5 score at study end.

Patient subgroups were created based on the self-reported presence of current medical conditions at the screening visit of each study. Information on these conditions was captured in the electronic case report form (eCRF), which included pre-defined medical condition categories that were subsequently grouped into comorbid condition subgroups. These subgroups were upper respiratory (allergic rhinitis/hay fever, sinusitis, nasal polyps), psychopathologies (anxiety, depression, mood changes, sleep disorders), cardiovascular (arrythmia, cardiac failure, cardiomyopathy, coronary artery disease, hypertension, hyperglycemia, cerebrovascular disorder, thrombophlebitic event), GERD, diabetes mellitus, and obesity (body mass index > 30).

A separate post hoc analysis of conditions potentially associated with long-term OCS use was conducted in patients who were OCS-dependent, defined as patients with evidence of long-term OCS usage (treatment with OCS for ≥ 50% of the year or medium-dose [6–12 mg/day] or high-dose [> 12 mg/day] OCS use for > 6 months prior to baseline visit) who were receiving OCS at baseline. Data from these patients were analyzed according to the presence of the following OCS-related conditions at screening, which were adrenal-related (adrenal suppression, Cushing’s syndrome, moon face), psychopathologies (anxiety, depression, mood changes, sleep disorders), eye-related (glaucoma, cataract), osteoporosis/bone fractures (bone fractures, osteoporosis), bruising, and weight gain.

### Statistical analysis

All analyses were conducted in the intent-to-treat population, which included all randomized patients who received ≥ 1 dose of study medication. Patients were analyzed based on the treatment received. For the purposes of the analysis, all doses of mepolizumab used during the four studies were combined into a single treatment group.

The rate of clinically significant exacerbations was analyzed using a negative binomial generalized linear model with a log-link function, including log of time on treatment as an offset variable. Change from baseline in SGRQ total score was analyzed using analysis of covariance. Change from baseline in ACQ-5 score and change from baseline in pre-bronchodilator FEV_1_ were analyzed using a mixed model repeated measures (MMRM) analysis. All model-based analyses included study ID, treatment group, region (European Union [EU], Europe [non-EU], South America, United States, rest of world), number of exacerbations in the prior year (0, 1, or 2 vs 3 vs ≥ 4), baseline maintenance OCS therapy (OCS vs no OCS), and baseline % predicted FEV_1_ (except change from baseline in pre-bronchodilator FEV_1_) as fixed effects. For change from baseline analyses (SGRQ, ACQ-5, and FEV_1_), the corresponding baseline value was also included as a covariate. For MMRM analyses (ACQ-5 and FEV_1_) time (fitted as a categorical variable), visit by baseline, and visit by treatment group interactions were also included in the model.

**Fig. 1:**
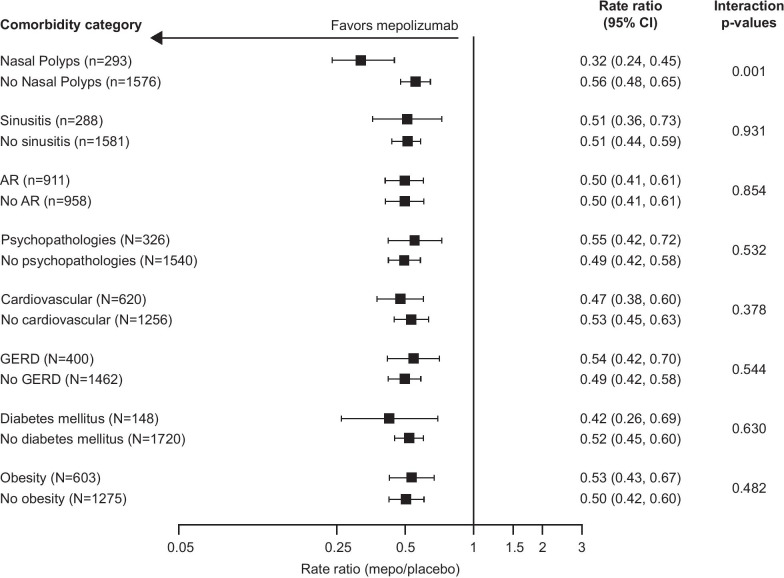
Rate of clinically significant exacerbations by comorbidity category. The rate of clinically significant exacerbations was analyzed using a negative binomial generalized linear model with a log-link function, including log of time on treatment as an offset variable. p-interaction < 0.1. *AR* allergic rhinitis/hay fever; *CI* confidence interval; *GERD* gastroesophageal reflux disease; *mepo* mepolizumab

**Fig. 2:**
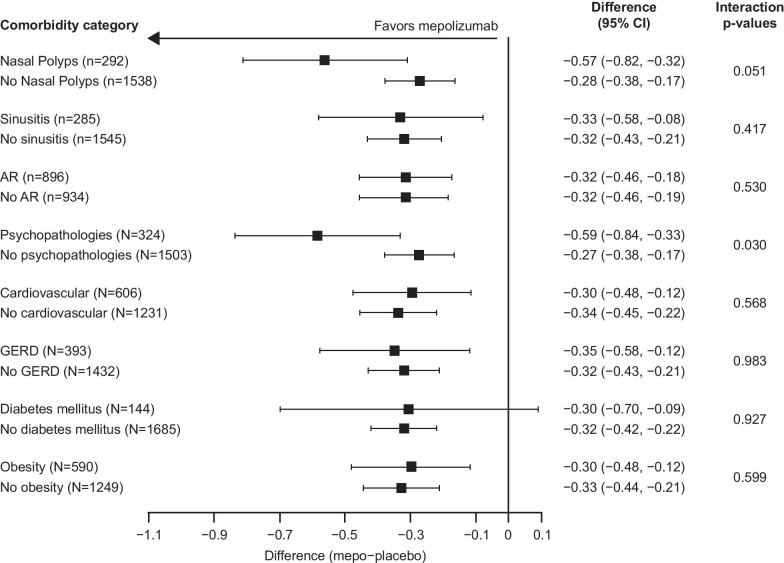
Change from baseline in ACQ-5 score at Week 24 by comorbidity category. Change from baseline in ACQ-5 score was analyzed using a MMRM analysis. The currently accepted minimum clinically important difference for ACQ-5 score is 0.5 points (established in adults with symptomatic asthma) [38]. p-interaction < 0.1. *ACQ*, Asthma Control Questionnaire; *AR* allergic rhinitis/hay fever; *CI* confidence interval; *GERD* gastroesophageal reflux disease; *mepo* mepolizumab; *MMRM* mixed model repeated measures

**Fig. 3:**
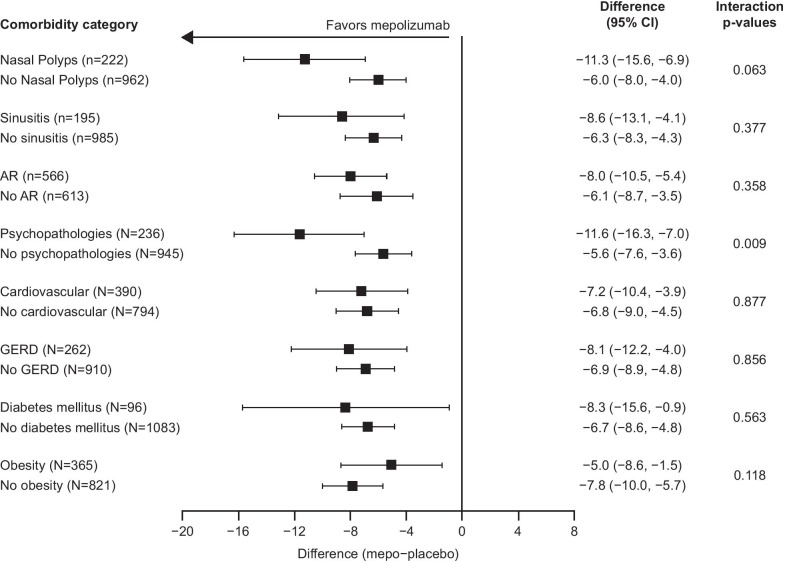
Change from baseline in SGRQ total score at Week 24 by comorbidity category. Change from baseline in SGRQ total score was analyzed using analysis of covariance. The currently accepted minimum clinically important difference for SGRQ is 4 units (established in an average population of patients with chronic obstructive pulmonary disease) [25]. p-interaction < 0.1. *AR* allergic rhinitis/hay fever; *CI* confidence interval; *GERD* gastroesophageal reflux disease; *mepo* mepolizumab; *SGRQ* St George’s Respiratory Questionnaire

**Fig. 4:**
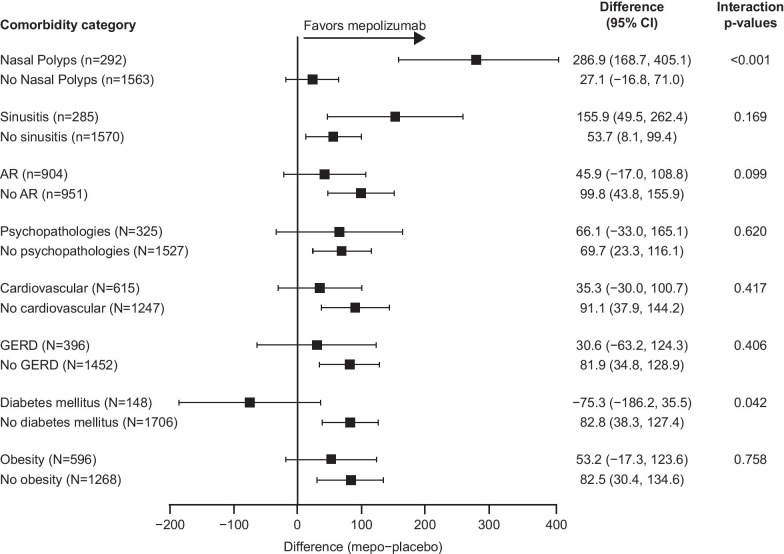
Change from baseline in pre-bronchodilator FEV_1_ (mL) at Week 24 by comorbidity category. Change from baseline in pre-bronchodilator FEV_1_ was analyzed using a MMRM analysis. p-interaction < 0.1. *AR* allergic rhinitis/hay fever; *CI* confidence interval; *FEV*_*1*_ forced expiratory volume in 1 s; *GERD* gastroesophageal reflux disease; *mepo* mepolizumab; *MMRM* mixed model repeated measures

Interaction p-values at a 10% threshold level were calculated to assess treatment effect across the subgroups with and without comorbidities. Interaction p-values (p < 0.1) are shown on Figs. 1, 2, 3 and 4. Interaction p-values were obtained from separate negative binomial regression models (rate of clinically significant exacerbations) or separate MMRM (for all other endpoints) with covariates as listed above but also including comorbidity category. For clinically significant exacerbations and SGRQ, the interaction term was comorbidity category by treatment group. For change from baseline in ACQ and pre-bronchodilator FEV_1_ analyses, the terms were visit by baseline, visit by treatment group, visit by comorbidity category, treatment by comorbidity category and treatment by visit by comorbidity category. Study ID was added as a fixed effect across all endpoints.

For DREAM, which collected ACQ-6 data, ACQ-5 scores were created using the first five elements of the ACQ-6.

## Results

### Patient population

In total, 1878 patients received ≥ 1 dose of either placebo (n = 689) or mepolizumab (n = 1189) during the DREAM, MENSA, SIRIUS, and MUSCA trials. Baseline demographics and clinical characteristics were similar between studies, with the exception of OCS usage in the SIRIUS study, where all patients were required to have been in receipt of maintenance OCS therapy at baseline (Table 1). At baseline, 1102 (59%) patients reported at least one upper airway comorbidity, with allergic rhinitis being the most common (Table 2). Additionally, 620 (33%) patients reported at least one cardiovascular condition, 603 (32%) patients reported obesity, and 400 (21%) patients reported gastroesophageal reflux (Table 2). Psychopathologies were reported by 326 (17%) patients, diabetes mellitus by 148 (8%) patients, and vocal cord dysfunction by 14 (< 1%) patients (Table 2). Of 544 (29%) OCS-dependent patients, 136 (25%) had psychopathologies at screening, 108 (20%) had osteoporosis/bone fractures, and 91 (17%) reported weight gain (Table 3).

**Table 1:** Baseline demographics and clinical characteristics

	DREAM (N = 616)	MENSA (N = 576)	SIRIUS (N = 135)	MUSCA (N = 551)	Total (N = 1878)
Age, years, mean (SD)	48.6 (11.3)	50.1 (14.3)	49.9 (12.3)	50.9 (13.5)	49.8 (13.0)
Female, n (%)	387 (63)	329 (57)	74 (55)	325 (59)	1115 (59)
BMI, kg/m^2^, mean (SD)	28.5 (5.95)	27.8 (5.83)	28.7 (6.01)	28.2 (6.40)	28.2 (6.06)
Duration of asthma, years, mean (SD)	19.1 (14.3)	19.9 (13.8)	18.7 (13.1)	19.5 (14.8)	19.5 (14.2)
Number of exacerbations in the previous year, n (%)					
≤ 2	286 (46)	246 (43)	67 (50)	357 (65)	956 (51)
3	154 (25)	141 (24)	20 (15)	96 (17)	411 (22)
≥ 4	176 (29)	189 (33)	48 (36)	98 (18)	511 (27)
Receiving maintenance OCS therapy at baseline, n (%)	188 (31)	144 (25)	135 (100)	131 (24)	598 (32)
% predicted pre-bronchodilator FEV_1_, mean (SD)	59.7 (15.89)	61.0 (17.99)	58.7 (17.75)	58.6 (16.04)	59.7 (16.75)
Blood eosinophil count, cells/µL, geometric mean (SD logs)	260 (0.957)	300 (0.950)	250 (1.081)	340 (0.943)	290 (0.966)

*BMI* body mass index, *FEV*_*1*_ forced expiratory volume in 1 s, *OCS* oral corticosteroids, *SD* standard deviation

**Table 2:** Patient co-morbidities across DREAM, MENSA, SIRIUS, and MUSCA

	Patients (N = 1878)
Any condition, n (%)	1569 (84)
Upper respiratory, n (%)	
Any condition	1102 (59)
Allergic rhinitis or hay fever	911 (49)
Sinusitis	288 (15)
Nasal polyps	293 (16)
Cardiovascular, n (%)	
Any condition	620 (33)
Arrythmia	49 (3)
Cardiac failure	13 (< 1)
Cardiomyopathy	4 (< 1)
Coronary artery disease	48 (3)
Hypertension	560 (30)
Hyperglycemia	48 (3)
Cerebrovascular disorder	7 (< 1)
Thrombophlebitic event	4 (< 1)
Obesity^a^, n (%)	603 (32)
Gastroesophageal reflux, n (%)	400 (21)
Psychopathologies, n (%)	
Any condition	326 (17)
Anxiety	141 (8)
Depression	149 (8)
Mood changes	78 (4)
Sleep disorders	158 (8)
Diabetes mellitus, n (%)	148 (8)
Vocal cord dysfunction, n (%)	14 (< 1)

*BMI* body mass index

^a^Defined as BMI > 30 kg/m^2^

**Table 3:** Patient OCS-dependence comorbidities in OCS-dependent patients.^a^

Diagnosis description	Patients (N = 544)
Any condition, n (%)	291 (53)
Psychopathologies, n (%)	
Any condition	136 (25)
Anxiety	55 (10)
Depression	55 (10)
Mood changes	40 (7)
Sleep disorders	68 (13)
Osteoporosis/bone fractures, n (%)	
Any condition	108 (20)
Bone fractures	5 (< 1)
Osteoporosis	107 (20)
Weight gain, n (%)	91 (17)
Adrenal-related, n (%)	
Any condition	59 (11)
Adrenal suppression	18 (3)
Cushing’s syndrome	20 (4)
Moon face	41 (8)
Bruising, n (%)	59 (11)
Eye-related, n (%)	
Any condition	54 (10)
Glaucoma	12 (2)
Cataract	44 (8)

*OCS* oral corticosteroids

^a^Patients who had treatment with continuous or near-continuous (at least half of the year) oral corticosteroids

### Primary endpoint

In the combined intent-to-treat population, the rate of clinically significant exacerbations was reduced by 49% with mepolizumab versus placebo (rate ratio [95% CI] 0.51 [0.45, 0.59]). This improvement was seen regardless of comorbid upper respiratory comorbidity status, with reductions of 68% and 44% in patients with and without nasal polyps, 49% in patients both with and without sinusitis, and 50% in patients both with and without allergic rhinitis/hay fever, respectively (Fig. 1). Reductions in the rate of clinically significant exacerbations with mepolizumab versus placebo were also seen across all other comorbidity subgroups, ranging between 45 and 58% in the psychopathologies and diabetes mellitus subgroups, respectively (Fig. 1). Reductions in the rate of clinically significant exacerbations ranging between 16 and 64% were also shown across OCS-dependent comorbidity categories (Additional file 1: Fig. 1), although rates in the adrenal-related, psychopathologies, and eye-related comorbidity subgroups were non-estimable due to an insufficient number of patients.

### Secondary endpoints

In the combined intent-to-treat population, there was a 0.32-point improvement in ACQ-5 score with mepolizumab versus placebo. Again, improvements were shown both in patients with and in those without nasal polyps, sinusitis, or allergic rhinitis/hay fever (Fig. 2). Improvements in ACQ-5 score were seen across all other comorbidity subgroups, with the smallest improvement in the no psychopathologies subgroup (Fig. 2). A wide confidence interval (CI) was observed for patients with diabetes mellitus compared with other comorbidities, likely owing to the comparatively small sample size of this patient subgroup. Improvements in ACQ‑5 score were also seen across all OCS-dependent comorbidity categories, except the adrenal-related comorbidities subgroup (Additional file 1: Fig. 2).

In the combined intent-to-treat population, mepolizumab-treated patients demonstrated a 6.9-point improvement in SGRQ total score compared with placebo-treated patients. Improvements in SGRQ total score were seen in the nasal polyps subgroup (11.3-point improvement) and in the no nasal polyps subgroup (6.0-point improvement), in the sinusitis subgroup (8.6-point improvement) and in the no sinusitis subgroup (6.3-point improvement), and in the allergic rhinitis/hay fever subgroup (8.0-point improvement) and in the no allergic rhinitis/hay fever subgroup (6.1-point improvement) (Fig. 3). Improvements in SGRQ total score with mepolizumab versus placebo were consistent across all other comorbidity subgroups, ranging from a 5.0-point improvement in the obesity subgroup to an 11.6-point improvement in the psychopathologies subgroup (Fig. 3). Similar to ACQ-5, a wide CI was seen in the diabetes mellitus subgroup compared with other comorbidities. Improvements in SGRQ total score were also seen across all OCS-dependent comorbidity subgroups, except the eye-related subgroup (Additional file 1: Fig. 3).

In the combined intent-to-treat population there was a 71.8 mL improvement in pre-bronchodilator FEV_1_ with mepolizumab versus placebo. Increases in pre-bronchodilator FEV_1_ with mepolizumab compared with placebo were greater in patients with nasal polyps than in those without and in those with sinusitis than in those without (Fig. 4). Greater improvements in pre-bronchodilator FEV_1_ with mepolizumab versus placebo were also seen in patients without allergic rhinitis/hay fever compared with patients with this comorbidity (Fig. 4). Improvements in pre-bronchodilator FEV_1_ were seen in all subgroups with the exception of the diabetes mellitus subgroup (Fig. 4); the smallest improvement was noted in the GERD subgroup and the greatest in the no cardiovascular comorbidity subgroup. Improvements in pre-bronchodilator FEV_1_ were also seen across all OCS-dependent comorbidity subgroups, except the eye-related subgroup (Additional file 1: Fig. 4).

## Discussion

In this post hoc meta-analysis of four Phase IIb/III, randomized, placebo-controlled studies, mepolizumab demonstrated consistent improvements versus placebo in the rate of clinically significant exacerbations, health-related quality of life, and asthma control, independent of patients’ comorbidities; improvements were also noted in lung function. The comparable efficacy of mepolizumab in patients with a range of airway-related, non-airway-related, and iatrogenic comorbidities demonstrates the suitability of mepolizumab treatment in patients with severe eosinophilic asthma and such comorbid conditions.

The effect of mepolizumab in patients with severe asthma and comorbidities has not, to date, been comprehensively investigated. In this analysis, comorbidity subgroups were selected based on those comorbidities that are most commonly found in patients with severe asthma and are most impactful in terms of their effect on symptom control and disease management. In particular, patients with asthma and upper respiratory comorbidities such as chronic rhinosinusitis have worse outcomes than those with asthma alone.

Targeting type 2 inflammation via interleukin-5 inhibition has been associated with clinical benefits in patients with asthma and upper airway comorbidities [11, 23, 26]. Separately, mepolizumab has been shown to be efficacious and well tolerated in patients with nasal polyps, who often have asthma as a comorbidity [23, 27]. Other concomitant asthma comorbidities or traits that have been shown to be associated with poorer asthma outcomes in patients versus those with just asthma are psychopathologies, such as anxiety, depression, and sleep disorders, obesity, and GERD [28–32].

Interestingly, the subgroups with nasal polyps in the current analysis showed significantly improved benefits with mepolizumab versus placebo compared with their counterparts without this upper respiratory comorbidity. These benefits were seen in the rate of clinically significant exacerbations, change from baseline in ACQ-5 score and SGRQ total score, and the change from baseline in pre-bronchodilator FEV_1_. These findings are consistent with an effect of mepolizumab in reducing the additional symptomatic and health-related quality of life impact of severe nasal polyposis [11]. For patients with sinusitis and those with allergic rhinitis/hay fever no significant additional benefits in change from baseline in SGRQ total score with mepolizumab versus placebo were observed when compared with patients without these comorbidities. Our analysis contrasts with results from a recent single-center retrospective study, which demonstrated that in patients receiving mepolizumab for severe eosinophilic asthma, those with eosinophilic chronic rhinosinusitis achieved greater improvement in clinical variables compared with those without [33].

Another interesting effect was the significant additional improvement in ACQ-5 score and SGRQ total score in the psychopathologies subgroup compared with the no psychopathologies subgroup. Previous studies have shown an association between conditions such as depression and anxiety and a patient’s ability to self-manage their asthma, with some patients adhering poorly to their medication regimens [34]. As a result, patients with depression and anxiety often have worse asthma symptom control and poorer quality of life than those with asthma alone [35, 36]. Improvements in asthma symptom control (as shown by improved ACQ-5 score) and health-related quality of life (as shown by improved SGRQ total score) shown in this analysis may be partially explained by patients attending visits every 4 weeks and/or improved adherence to treatment as a result of being part of a monitored clinical trial. Separately, it is also possible that biologic treatment such as mepolizumab may overcome issues of non-adherence and poor administration technique associated with other therapies that patients may have been in receipt of previously (such as inhaler-based treatment), resulting in disproportionate improvements in quality of life for those with psychopathologies.

In this analysis, we observed no additional significant clinical benefits with mepolizumab versus placebo in the no obesity subgroup compared with those in the obesity subgroup. These findings are in contrast with a post hoc analysis of MENSA and MUSCA, which found a trend for smaller clinical improvements with mepolizumab versus placebo in patients in the highest body weight and body mass index categories [37], which has also been seen with other biologic treatments for severe asthma [38, 39]. One explanation for the smaller clinical improvements seen in patients in the highest body weight and body mass index categories could be airway restriction due to mechanical factors in the pleural cavity [39].

Due to the low numbers of patients with diabetes, accurate comparisons between subgroups were difficult, with large CIs for each of the endpoints. The rate of clinically significant exacerbations was slightly lower for those with diabetes than without, the ACQ-5 score appeared similar, SGRQ total score was slightly higher in those with diabetes versus those without, and the change from baseline in pre-bronchodilator FEV_1_ was lower in those with diabetes versus those without. Given that there was no consistent reduction or enhancement of clinical benefit and due to the limited number of patients it is difficult to make any firm conclusions on the impact of this comorbidity on mepolizumab efficacy.

Mepolizumab has demonstrated clinically important OCS-sparing effects in patients with severe eosinophilic asthma [19]. Given that OCS-dependent patients with severe asthma may be eligible for mepolizumab treatment, this analysis also investigated the efficacy of mepolizumab in patients with severe eosinophilic asthma and comorbidities related to the long-term use of OCS. Although most of the comorbidity subgroups were limited by patient numbers, reductions in the rate of clinically significant exacerbations ranging between 16 and 64% were seen in all subgroups in which there were sufficient patient numbers. Improved ACQ-5 scores and SGRQ total scores were also seen in all comorbidity subgroups with the exception of the adrenal-related and eye-related subgroups, respectively. Finally, improvements in pre-bronchodilator FEV_1_ were also seen in all but the eye-related subgroup; it should be noted that the CIs were large in the pre-bronchodilator FEV_1_ subgroups, which may be due to small patient numbers. As over 90% of OCS-dependent patients with severe asthma experience comorbidities associated with long-term OCS exposure [4], these findings are of particular importance for clinicians treating patients who are currently using OCS but are eligible for a switch to mepolizumab.

This meta-analysis of four similar studies provided a large patient sample in which to determine the effect of mepolizumab across patients with various comorbidities. However, there were a number of limitations. First, this analysis was conducted post hoc, and this should be taken into consideration when interpreting the findings. Moreover, the number of patients varied greatly between comorbidity subgroups, with the smallest patient numbers seen in the diabetes, psychopathologies, and GERD subgroups. The placement into subgroups was based on patient self-reporting of the comorbid conditions, which is less accurate than determining diagnosis as part of the study, and it is important to note that the severity of each comorbidity was not analyzed within this study. Furthermore, the subgroup analyses were not adjusted for potential confounding effects (for example, blood eosinophil counts). Additionally, while the studies were similar with respect to patient population and standard of care therapy, and study identifier was included in the meta-analysis as a fixed effect to account for between-study variability, there were several differences that should be considered, including several differences in the study inclusion criteria, the length of treatment and the administration type and/or dose of mepolizumab included. Also, patients with multiple comorbidities were not enrolled in the individual studies, based on the study inclusion and exclusion criteria. This likely excluded many patients such as those with clinically important lung conditions, liver disease, malignancies, and severe cardiovascular disease. Ongoing and planned observational studies will hopefully provide real-world data on the impact of mepolizumab treatment in these patients. Finally, there was no investigation of the safety of mepolizumab across the comorbidity subgroups, although mepolizumab was shown to be well tolerated in the four parent studies [19–22]. Despite these limitations, the analysis provides important information for clinicians regarding the efficacy of mepolizumab in patients with severe eosinophilic asthma who also have common and impactful comorbidities.

In summary, results from this post hoc meta-analysis of four Phase IIb/III clinical trials indicate that mepolizumab treatment is associated with similar improvements in exacerbation rate, asthma control, health-related quality of life and lung function in patients with severe eosinophilic asthma and self-reported comorbid upper airway disease or other comorbidities. These data suggest that mepolizumab is of clinical benefit to provide targeted treatment and help reduce disease burden in those individuals with severe eosinophilic asthma with comorbid conditions.

## Supplementary Information

**Additional file 1: Table 1.** Key inclusion criteria for the four pivotal mepolizumab studies. **Figure 1.** Rate of clinically significant exacerbations by OCS-dependence comorbidity category. **Figure 2.** Change from baseline in ACQ-5 score at Week 24 by OCS-dependence comorbidity category. **Figure 3.** Change from baseline in SGRQ total score at study end by OCS-dependence comorbidity category. **Figure 4.** Change from baseline in pre-bronchodilator FEV_1_ (mL) at Week 24 by OCS-dependence comorbidity category.

## Data Availability

Anonymized individual participant data from the studies listed within this publication and their associated documents can be requested for further research from www.clinicalstudydatarequest.com.

## Abbreviations

ACQ: Asthma Control Questionnaire
AR: Allergic rhinitis
CI: Confidence interval
eCRF: Electronic case report form
EU: European Union
FEV_1_: Forced expiratory volume in 1 s
GERD: Gastroesophageal reflux disease
MMRM: Mixed model repeated measures
OCS: Oral corticosteroid
NP: Nasal polyps
SD: Standard deviation
SGRQ: St George’s Respiratory Questionnaire
