# Supplementary material for: Mepolizumab improves clinical outcomes in patients with severe asthma and comorbid conditions
Source: Respir Res. 2021 Jun 7;22:171. doi: 10.1186/s12931-021-01746-4 (PMC8182929; doi:10.1186/s12931-021-01746-4)
Supplement: Supplementary file 1 — Additional file 1: Table 1. Key inclusion criteria for the four pivotal mepolizumab studies. Figure 1. Rate of clinically significant exacerbations by OCS-dependence comorbidity category. Figure 2. Change from baseline in ACQ-5 score at Week 24 by OCS-dependence comorbidity category. Figure 3. Change from baseline in SGRQ total score at study end by OCS-dependence comorbidity category. Figure 4. Change from baseline in pre-bronchodilator FEV1 (mL) at Week 24 by OCS-dependence comorbidity category. [file 12931_2021_1746_MOESM1_ESM.docx]

**Additional File for:**

**Mepolizumab improves clinical outcomes in patients with severe asthma and comorbid conditions**

Peter G. Gibson, Charlene M. Prazma, Geoffrey L. Chupp, Eric S. Bradford, Mark Forshag, Stephen A. Mallett, Steve W. Yancey, Steven G. Smith, Elisabeth H. Bel

**Table of Contents**

[Additional Table 1 2](#_Toc72769316)

[Additional Figure 1 3](#_Toc72769317)

[Additional Figure 2 4](#_Toc72769318)

[Additional Figure 3 5](#_Toc72769319)

[Additional Figure 4 6](#_Toc72769320)

Additional Table 1. Key inclusion criteria for the four pivotal mepolizumab studies.

|  | **DREAM** | **MENSA** | **SIRIUS** | **MUSCA** |
| --- | --- | --- | --- | --- |
| **Key inclusion criteria** | | | | |
| ***Age group*** | ≥12 years | ≥12 years | ≥12 years | ≥12 years |
| ***Exacerbation history*** | ≥2 exacerbations requiring corticosteroid treatment in previous 12 months | ≥2 exacerbations requiring corticosteroid treatment in previous year | Not required | ≥2 exacerbations requiring corticosteroid treatment in previous year |
| ***Sputum eosinophil count*** | Historical or baseline 3%^a^ | Not required | Not required | Not required |
| ***Peripheral blood eosinophil count*** | Historical count (12 months) ≥300 cells/µL | Historical count (12 months) ≥300 cells/µL OR baseline count ≥150 cells/µL | Historical count (12 months) ≥300 cells/µL OR baseline count ≥150 cells/µL | Historical count (12 months) ≥300 cells/µL OR baseline count ≥150 cells/µL |
| ***Baseline treatments^b^*** | High-dose ICS ± OCS + controller in previous 12 months | High-dose ICS ± OCS in previous 12 months + controller for ≥3 successive months in previous 12 months | High-dose ICS + OCS for ≥6 months + controller for ≥3 successive months in previous 12 months | High-dose ICS ± OCS in previous 12 months + controller for ≥3 successive months in previous 12 months |
| ***Lung function*** | Pre-bronchodilator FEV_1_ <80% predicted OR peak flow diurnal variability of >20% on ≥3 days during run-in | Pre-bronchodilator FEV_1_ <80% predicted | Pre-bronchodilator FEV_1_ <80% predicted | Pre-bronchodilator FEV_1_ <80% predicted |

^a^Eosinophilic inflammation was defined by one of several criteria, of which sputum eosinophil and peripheral blood cell count were two possibilities. Patients were required to meet one of these criteria to be included in the study; ^b^controller medication: LABA, LTRA, or theophylline.
FEV_1_, forced expiratory volume in 1 second; ICS, inhaled corticosteroid; LABA, long-acting β_2_-agonist; LTRA, leukotriene receptor antagonists; OCS, oral corticosteroid.

Additional Figure 1. Rate of clinically significant exacerbations by OCS-dependence comorbidity category.


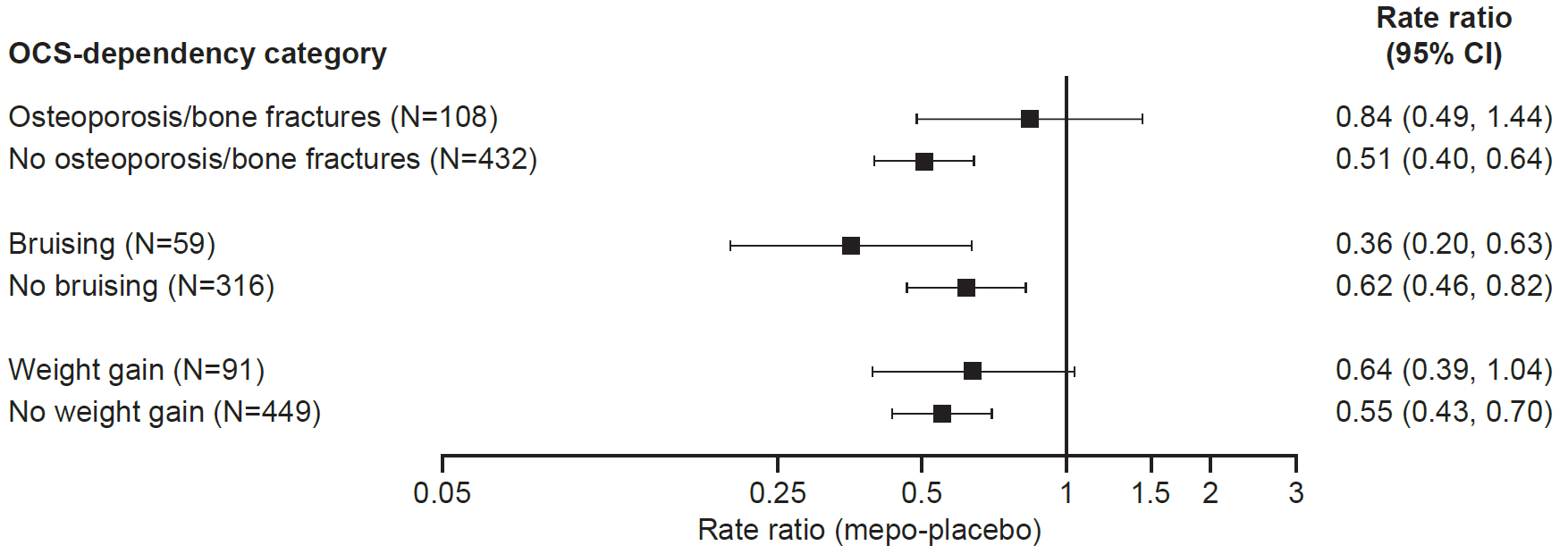


The rate of clinically significant exacerbations was analyzed using a negative binomial generalized linear model with a log-link function, including log of time on treatment as an offset variable. Horizontal bars indicate 95% confidence intervals for the mepolizumab/placebo rate ratio. Adrenal-related, psychopathologies, and eye-related analyses not performed due to insufficient number of patients. CI, confidence interval; OCS, oral corticosteroid.

Additional Figure 2. Change from baseline in ACQ-5 score at Week 24 by OCS-dependence comorbidity category.


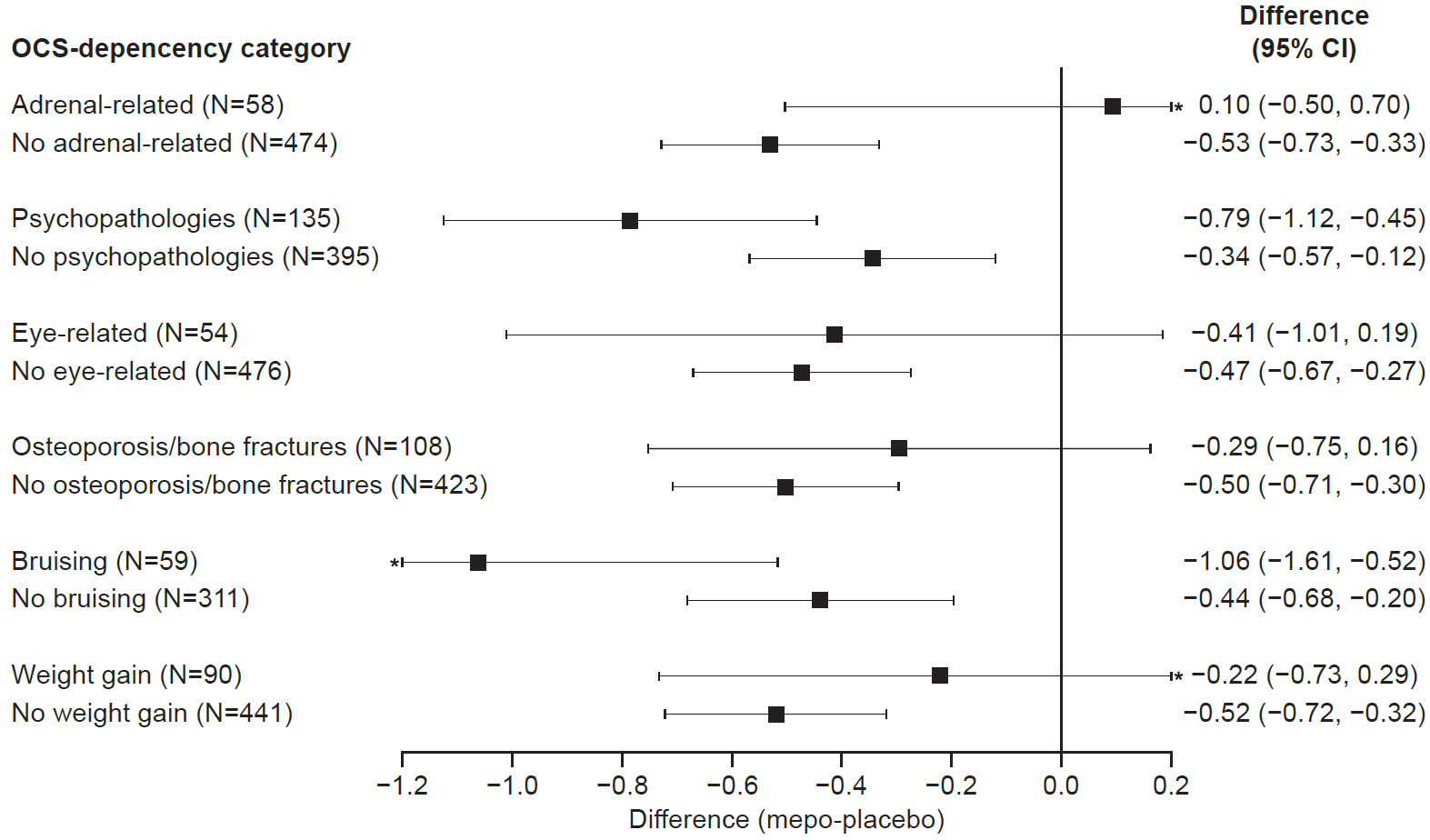


Change from baseline in ACQ-5 score was analyzed using a MMRM analysis. Horizontal bars indicate 95% confidence intervals for the treatment difference between mepolizumab and placebo. *Denotes lower/upper confidence interval extends beyond the range of the x axis. ACQ, Asthma Control Questionnaire; CI, confidence interval; MMRM, mixed model repeated measures;

OCS, oral corticosteroid.

Additional Figure 3. Change from baseline in SGRQ total score at study end by OCS-dependence comorbidity category.


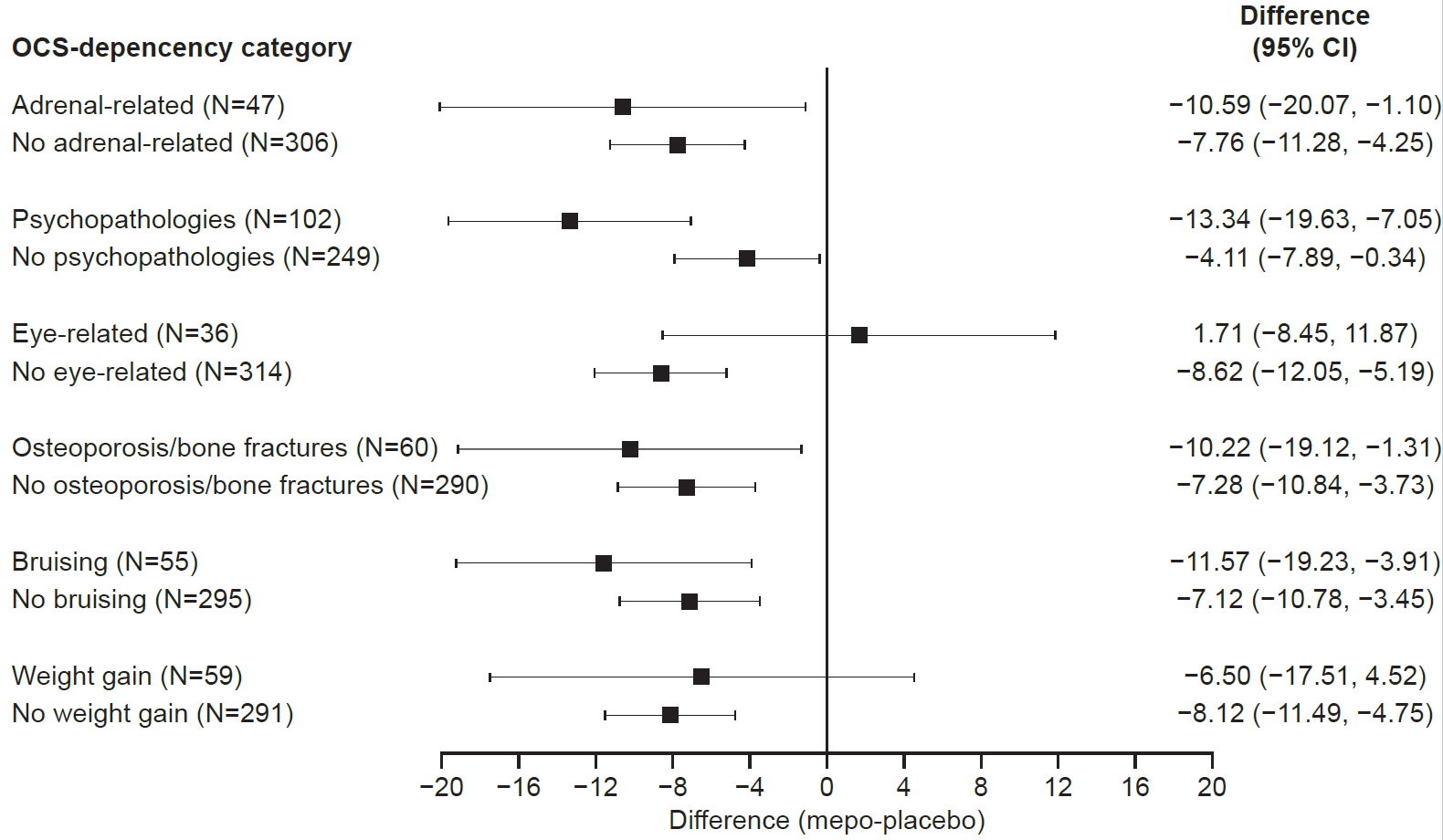


Change from baseline in SGRQ total score was analyzed using analysis of covariance. Horizontal bars indicate 95% confidence intervals for the treatment difference between mepolizumab and placebo. CI, confidence interval; OCS, oral corticosteroid; SGRQ, St George’s Respiratory Questionnaire.

Additional Figure 4. Change from baseline in pre-bronchodilator FEV_1_ (mL) at Week 24 by OCS-dependence comorbidity category.


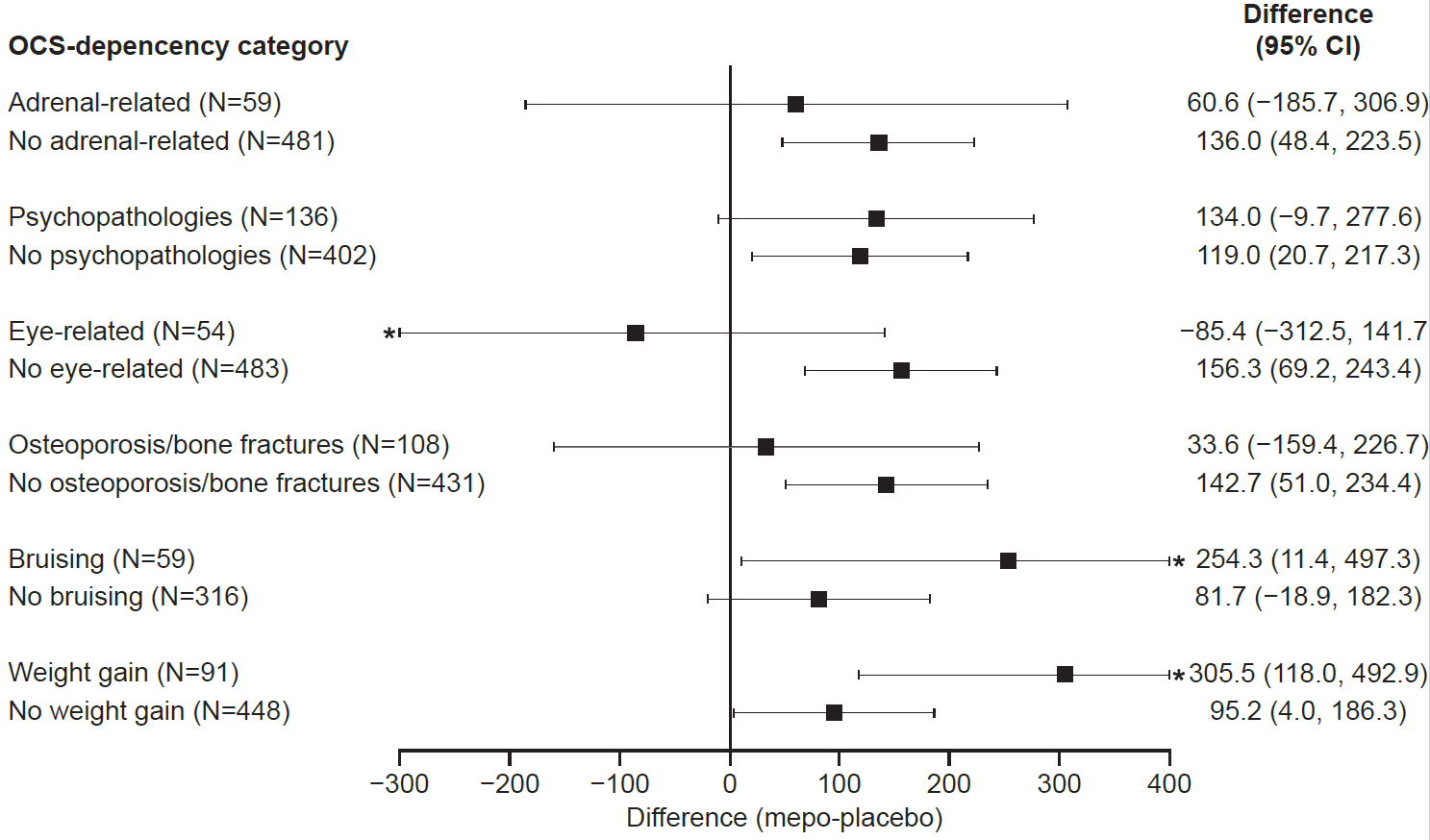


Change from baseline in pre-bronchodilator FEV_1_ was analyzed using a MMRM analysis. Horizontal bars indicate 95% confidence intervals for the treatment difference between mepolizumab and placebo. *Denotes lower/upper confidence interval extends beyond the range of the x axis. CI, confidence interval; FEV_1_, forced expiratory volume in 1 second; MMRM, mixed model repeated measures; OCS, oral corticosteroid.
